# Neurosarcoidosis: A Rare Presentation as a Seizure

**DOI:** 10.7759/cureus.40227

**Published:** 2023-06-10

**Authors:** Saikiran Mandyam, Sunilkumar Sibyala, Preethi Dasarathan, Yagnapriya Chirrareddy, Pavan Kumar Reddy Kalluru

**Affiliations:** 1 Graduate Medical Education (GME) Internal Medicine, Southeast Health Medical Center, Dothan, USA; 2 Internal Medicine, Sri Venkateswara Medical College, Tirupati, IND; 3 Internal Medicine, Sri Padmavathi Medical College, Tirupati, IND

**Keywords:** aseptic meningitis, leptomeningeal enhancement, high-dose steroids, seizure, neurosarcoidosis

## Abstract

Neurosarcoidosis is a rare disorder that can develop in patients with a history of sarcoidosis or can develop even when sarcoidosis is not diagnosed. It is a granulomatous disease of the nervous system that causes different neurological disorders based on its location. However, diagnosing neurosarcoidosis remains a challenge as it mimics many other neurological disorders and does not have any biochemical markers of high specificity. A tissue-proven biopsy is the gold standard but is difficult to obtain in neurological illnesses. Thus, diagnosis is established based on the clinical syndrome and imaging, which mostly show meningeal/parenchymal lesion enhancement, in addition to the exclusion of other causes. Glucocorticoids, immunosuppressants, and anti-tumour necrosis factor (TNF) drugs are the mainstays of treatment. We discuss a case of neurosarcoidosis in a 52-year-old woman with a known history of sarcoidosis.

## Introduction

Neurosarcoidosis occurs when sarcoidosis, an idiopathic immune-mediated widespread multi-systemic inflammatory non-caseating granulomatous disease, affects the central and peripheral nervous systems. Central nervous system lesions can disrupt normal brain function and cause neurological symptoms, including seizures. A presumptive diagnosis of neurosarcoidosis is often made based on magnetic resonance imaging (MRI) and cerebrospinal fluid (CSF) analysis results in the appropriate clinical setting [1]. It can mimic infections and malignant aetiologies. Current management strategies have substantially improved patient survival and include glucocorticoids, immunosuppressive agents, and anti-tumour necrosis factor (TNF) drugs.

## Case presentation

**Figure 1:**
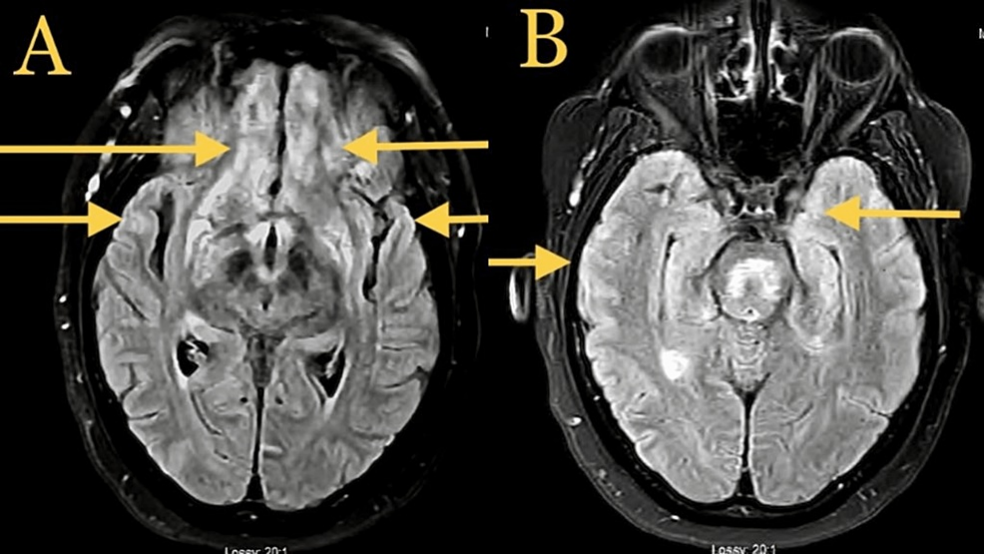
MRI head without IV contrast, axial view in FLAIR SPIR demonstrating diffuse nonspecific signal abnormality in frontal and temporal lobes indicated by yellow arrows

**Figure 2:**
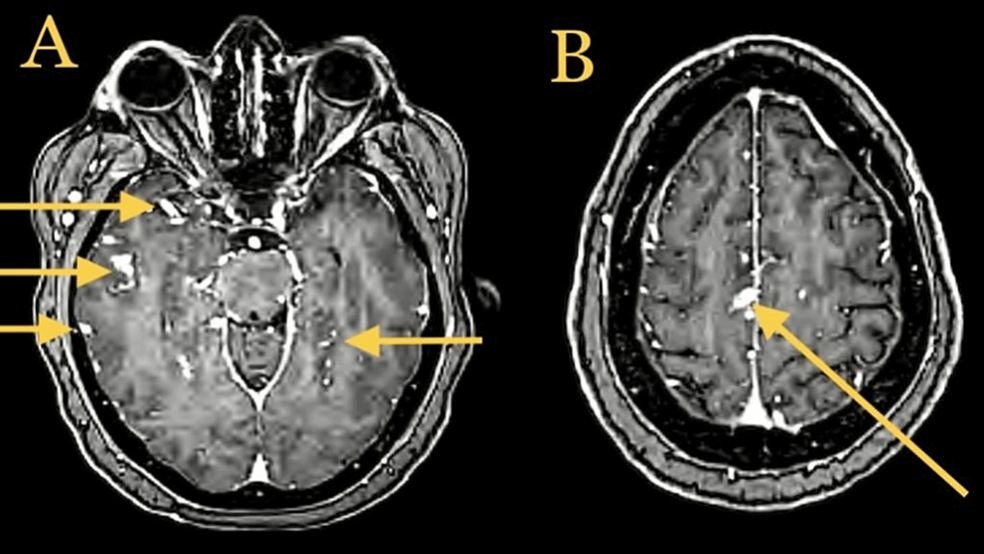
MRI head with contrast, axial T1 demonstrating abnormal diffuse enhancement (yellow arrows in image A), with the most significant area (4 mm × 7 mm) at the vertex (yellow arrow in image B)

A 52-year-old female with a past medical history of chronic obstructive pulmonary disease and sarcoidosis presented with new-onset generalized tonic-clonic seizures. Physical examination was negative for any acute abnormality; no gross neurological deficits were noted; pronator drift was negative; coordination, gait, sensory, and motor examinations were within normal limits. The skin examination was negative as well. An eye examination did not show any papules, conjunctivitis, uveitis, or episcleritis. No visual field defects were noted. The retinal exam was negative for macular edoema or vasculitis. The patient was loaded with levetiracetam. A computed tomography (CT) head without IV contrast was negative for an acute abnormality. The electroencephalogram (EEG) was negative for epileptiform waves, and the MRI head without comparison (Figure 1) showed some bitemporal and bifrontal abnormalities, as mentioned below. MRI of the brain with IV contrast (Figure 2) demonstrated leptomeningeal enhancement, which could be secondary to neurosarcoidosis. The differential diagnosis was broad, including malignancy, although neoplasms would not present as bilateral irregular enhancements. Serum calcium levels and angiotensin-converting enzyme (ACE) levels were within normal limits. CSF analysis demonstrated an aseptic meningitis pattern (Table 1). The patient was treated with high-dose IV steroids with methylprednisolone 250 mg twice daily per neurology recommendations for aseptic meningitis (CSF showing pleocytosis with lymphocytic predominance and elevated protein), likely a flare-up of neurosarcoidosis. Monitored in the hospital for two to three days, the patient did not have any further seizures and was subsequently discharged home with oral prednisone and levetiracetam 500 mg twice daily.

**Table 1:** Cerebrospinal fluid analysis CSF: cerebrospinal fluid; CSF ACE: CSF angiotensin converting enzyme; mg/dL: milligram per deciliter; U/L: units per liter; cells/µL: cells per microliter

CSF analysis	Normal range	Patients range
Oligoclonal bands	Negative	Negative
Meningitis panel	Negative	Negative
Culture	No growth	No growth
Glucose	40-70 mg/dL	55 mg/dL
Number of cells	< 5 cells/µL	100 cells/µL
Cell differential	Nil	84% lymphocytes, 9% macrophages, 6% neutrophils
Protein	15-45 mg/dL	145 mg/dL
CSF ACE	0-2.8 U/L	4.4 U/L

## Discussion

Neurosarcoidosis is an immune-mediated inflammatory disease characterised by noncaseating granulomas that affect both the central and peripheral nervous systems. In patients with known sarcoidosis who present with neurological symptoms, neurosarcoidosis should be considered a possible diagnosis, with infection and malignancy needing to be ruled out [2]. The clinical manifestations of neurosarcoidosis vary depending on the location of the granulomas. Cranial mononeuropathy can manifest as unilateral or bilateral facial nerve palsies, while optic neuropathy tends to have a worse prognosis than other cranial nerve neuropathies. Involvement of the hypothalamus directly can lead to endocrine abnormalities such as central diabetes insipidus or primary polydipsia. Hypercalcemia, caused by activated macrophages in the granuloma producing calcitriol, can also result in nephrogenic diabetes insipidus. Acute or subacute development of communicating or non-communicating hydrocephalus is possible, and an asymptomatic ventricular enlargement may be incidentally detected in imaging studies [3]. Granulomatous inflammation in a perivascular distribution can involve the brain and give rise to focal or generalised seizures. Peripheral neuropathies associated with neurosarcoidosis include mononeuropathy, mononeuritis multiplex, and generalised sensory, sensory-motor, and motor polyneuropathies [4]. Additionally, a less recognised neuropathic presentation is a disorder affecting tiny nerve fibres, characterised by the impaired perception of distal limb pain and temperature, often accompanied by paresthesias, hyperesthesia/dysesthesia, and autonomic dysfunction. In severe cases, these symptoms may extend to the torso.

The diagnosis of neurosarcoidosis is typically made based on clinical features, imaging findings, and histopathological examination, following the exclusion of other potential causes [2]. CSF analysis may reveal elevated total protein and white blood cell counts. However, the diagnostic utility of these markers is limited due to the possibility of minimal changes occurring in conditions such as multiple sclerosis or tumours. Glucose levels in the CSF can be normal or decreased. CSF ACE activity is a useful biochemical marker for probable neurosarcoidosis, mainly when diffuse enhancing lesions are observed on the brain or spinal MRI [5]. Nevertheless, CSF-ACE levels may also be increased in cases of infectious or carcinomatous meningitis. Neurosarcoidosis associated with aseptic meningitis cannot be distinguished from aseptic meningitis caused by infections like HIV, TB, or syphilis [6]. Although CSF-soluble interleukin-2 receptor levels can be elevated in CNS sarcoidosis, this test is not widely available or routinely used for diagnosing neurosarcoidosis. One study reported that levels exceeding 150 picograms/mL suggest neurosarcoidosis with a sensitivity of 61% and specificity of 93% compared to healthy controls and other inflammatory diseases such as multiple sclerosis and CNS vasculitis [7]. However, elevated levels can also be observed in the context of infection. MRI findings in neurosarcoidosis encompass a range of manifestations, including isolated mass lesions, diffuse intraparenchymal inflammatory lesions in the brain and spinal cord, leptomeningeal enhancement, hydrocephalus, and intracranial haemorrhage [8,9]. In a case series involving 29 patients with neurosarcoidosis, approximately 40% demonstrated meningeal enhancement and multiple white matter lesions on MRI [1]. It is worth noting that granulomatous meningeal masses can resemble meningiomas in imaging studies. In cases where there is a significant dural enhancement, IgG4-related pachymeningitis should be considered a diagnostic possibility [6]. While MRI and CSF studies are sensitive in detecting inflammation, they lack specificity, posing a clinical challenge in establishing a definite diagnosis of neurosarcoidosis [10]. Whole-body fluorodeoxyglucose positron emission tomography (FDG-PET) has proven helpful in evaluating patients with neurological manifestations suspected to be due to sarcoidosis [11]. In cases where patients with known systemic sarcoidosis and neurological involvement progressively deteriorate despite therapy, a biopsy may be necessary.

Treatment strategies for neurosarcoidosis primarily involve using corticosteroids as the first-line drug. Other immunosuppressive agents, including azathioprine, cyclophosphamide, and mycophenolate mofetil, have effectively treated neurosarcoidosis [12]. Studies have demonstrated positive outcomes in CNS improvement using anti-tumour necrosis factor therapy, such as infliximab, in treating neurosarcoidosis [13]. In cases where CNS mass lesions persist or enlarge despite optimal immunosuppressive therapy, resection may need to be considered.

## Conclusions

In conclusion, neurosarcoidosis is rare and complex and needs rapid diagnostic testing with appropriate imaging. Initiation of immunosuppressive therapy is the cornerstone of treating neurosarcoidosis, as spontaneous remissions are less likely and can be complicated by permanent neurological damage. Physicians should also be vigilant and have a low threshold for suspecting neurosarcoidosis in patients with neurological manifestations, including seizures.
